# Salmon Nasal Cartilage Proteoglycan Ameliorate Joint Pain and Cartilage Degradation by Regulating Catabolic and Anabolic Homeostasis in MIA-Induced Osteoarthritis

**DOI:** 10.3390/nu18010176

**Published:** 2026-01-05

**Authors:** Min Yu, So Eun Jo, Young Bae Son, Ye Jin Kim, Youngsik Seo, Sang Bae Han, Hyun Jin Kim, Seon Gil Do, Hanjoong Jo, Dong Ju Son

**Affiliations:** 1 College of Pharmacy, Chungbuk National University, 194-21 Osongsaengmyoung 1-ro, Osong-eup, Heungduk-gu, Cheongju 29160, Chungbuk, Republic of Korea; yumin0413@chungbuk.ac.kr (M.Y.); lovel5508@chungbuk.ac.kr (S.E.J.); yb@chungbuk.ac.kr (Y.B.S.); yejinee13@chungbuk.ac.kr (Y.J.K.); seoys@chungbuk.ac.kr (Y.S.); shan@chungbuk.ac.kr (S.B.H.); 2 R&D Center, Naturetech Inc., 450-86 Maebong-ro, Byeongcheon-myeon, Dongnam-gu, Cheonan 31257, Chungnam, Republic of Korea; hjmari@naturetech.co.kr (H.J.K.); sgildo@naturetech.co.kr (S.G.D.); 3 Wallace H. Coulter Department of Biomedical Engineering, Emory University and Georgia Institute of Technology, 1760 Haygood Drive NE, Atlanta, GA 30306, USA; hjo@emory.edu

**Keywords:** osteoarthritis, salmon nasal cartilage proteoglycan, inflammation, cartilage-degrading enzyme

## Abstract

Background/Objectives: Osteoarthritis (OA) is a pervasive chronic joint disease characterized by the triad of persistent articular cartilage degeneration, debilitating synovial inflammation, and sustained chronic pain. Although salmon nasal cartilage proteoglycan (SPG) is recognized for supporting joint health, the precise molecular mechanism underlying its effects during OA progression remains to be fully elucidated. This study evaluated the therapeutic efficacy of SPG using a monosodium iodoacetate (MIA)-induced mouse model. Methods: A total of 180 male C57BL/6J mice (six-week-old) were utilized, organized into three independent cohorts to analyze distinct analytical endpoints: (1) pain assessment, histology, and immunohistochemistry; (2) mRNA expression analysis for early-stage OA (Day 3); and (3) mRNA expression analysis for the late-stage OA (Day 28). All subjects received daily oral treatment via gavage, commencing 5 days prior to OA induction and continuing until the designated experimental termination points (either Day 3 or Day 28). Each cohort comprised five experimental groups (*n* = 10–12 per group): a saline-injected Sham group, an MIA-induced Control group, a positive comparator receiving celecoxib (CLX, 20 mg/kg/day), and two groups administered SPG at a dose of 50 or 100 mg/kg/day. Results: Our findings demonstrated that SPG, particularly at the 100 mg/kg dose, significantly mitigated joint pain symptoms, performing comparably to CLX. Histopathological assessments confirmed that SPG effectively preserved the structural integrity of the cartilage matrix and substantially reduced pathological damage, as evidenced by lower Mankin scores. Mechanistically, SPG treatment led to a marked downregulation of degradative enzymes, including matrix metalloproteinase-3 (MMP-3) and a disintegrin and metalloproteinase with thrombospondin motifs 4 (ADAMTS-4), while concurrently normalizing the levels of tissue inhibitors of metalloproteinases (TIMPs). Furthermore, SPG prevented the aberrant, over-compensatory expression of anabolic markers such as SRY-box transcription factor 9 (SOX-9), type II collagen alpha 1 chain (COL2A1), and aggrecan (ACAN) typically observed in the disease’s later stages. While SPG demonstrated a limited impact on broadly pro-inflammatory cytokine profiles, it specifically and significantly reduced interleukin-6 (IL-6) gene expression during the chronic phase. Conclusions: These results suggest that SPG serves as a promising natural agent that maintains articular homeostasis by balancing matrix metabolic pathways, positioning it as a scientifically validated functional food candidate for the management of joint health.

## 1. Introduction

Osteoarthritis (OA) is a highly prevalent and debilitating chronic disorder of the joints, primarily characterized by the irreversible deterioration of articular cartilage [1,2,3]. Beyond cartilage loss, the pathology encompasses subchondral bone changes, synovial inflammation, and persistent pain, leading to functional impairment. This condition severely impairs the quality of life; knee OA, in particular, is debilitating due to pain and mobility issues exacerbated by weight-bearing, which can even precipitate depression [4,5]. The core pathology of OA involves persistent joint inflammation and the irreversible deterioration of articular cartilage [6,7,8], with this cartilage loss representing the cardinal feature of the condition [9,10,11]. This degradation is primarily driven by the dysfunction of chondrocytes [12,13,14], the cells responsible for maintaining cartilage homeostasis [12,15].

Articular cartilage is primarily reliant on its extracellular matrix (ECM) for essential elasticity and resistance against mechanical loading, which fundamentally consists of type II collagen and the dominant proteoglycan, aggrecan [16]. The integrity of this vital ECM relies on a delicate equilibrium between anabolic (synthesis) and catabolic (degradation) activities [17]. Nevertheless, pro-inflammatory factors originating from the inflamed synovium stimulate chondrocytes, resulting in the excessive breakdown of the ECM and, ultimately, irreversible cartilage loss [18]. Therefore, a paramount therapeutic objective in OA management is to mitigate cartilage breakdown and actively preserve its structure [19,20].

Currently, clinical management largely relies on symptomatic relief (e.g., NSAIDs) and, ultimately, surgical joint replacement, as they fail to target the core mechanism of the disease [21]. Despite offering symptomatic relief, non-steroidal anti-inflammatory drugs (NSAIDs) pose significant restrictions on long-term, high-dose use due to their documented adverse events to the renal, cardiovascular, and gastrointestinal tracts [22,23]. The continued reliance on NSAIDs stems from a profound lack of comparably effective and safe agents [21]. Consequently, this unmet clinical need is driving the exploration of natural products as viable therapeutic avenues for OA [24]. Numerous functional ingredients with joint-health-promoting and cartilage-protective properties are gaining traction [24]. These include compounds such as glucosamine, *Boswellia serrata*, turmeric, olive leaf, and *Boletus edulis* extracts, representing promising avenues in the search for safer and more sustainable OA therapies [25,26,27].

The chum salmon (*Oncorhynchus keta)* is widely recognized as a premium source of diverse bioactive and nutritional substances. Beyond providing high biological value proteins, essential minerals, and vitamins, it is particularly rich in omega-3 polyunsaturated fatty acids (PUFAs), which are known for their cardioprotective and anti-inflammatory properties [28,29,30]. Furthermore, specific tissues yield specialized functional materials, such as polydeoxyribonucleotide (PDRN) from their milt [31]. Among these valuable components, salmon nasal cartilage proteoglycan (SPG) has emerged as a unique non-denatured proteoglycan isolated specifically from the nasal cartilage of this species. Its extraction via acetic acid offers distinct advantages, primarily the preservation of its critical biomolecular components and a simplified, cost-efficient, and scalable process [32]. Aggrecan is a cornerstone of articular cartilage’s ECM, and notably, SPG predominantly comprises this same pivotal proteoglycan [33]. This molecule is vital for cartilage function due to its capacity to bind with hyaluronic acid (HA), forming large aggregates that efficiently entrap water molecules [34]. These aggregates confer significant resistance to compressive forces from weight-bearing, providing a crucial “cushion-like” effect [35]. Beyond aggrecan, SPG’s intricate composition further includes other key glycosaminoglycans (GAGs) such as chondroitin sulfate, alongside essential non-collagenous proteins like chondronectin and link protein [32]. These components are instrumental in maintaining the structural integrity and stabilization of the cartilage ECM [36].

Recent scientific endeavors have extensively explored the diverse therapeutic efficacy of SPG, including its anti-inflammatory properties, wound healing capabilities, maintenance of skin condition, enhancement of cell growth, and regulation of metabolism, among others [37,38,39,40,41,42,43]. Of particular clinical relevance are its anti-arthritic effects [44,45].

Previous clinical trials have yielded encouraging findings; specifically, a daily oral dosage of 10 mg/kg of SPG, administered over 16 weeks, demonstrated a chondroprotective effect in subjects suffering from knee pain [44]. Furthermore, another investigation reported that a 12-week regimen of 10 mg/day oral SPG conferred pain relief and improved joint health in individuals with mild knee pain (predominantly Kellgren-Lawrence grade 0-II) [45].

While previous studies have highlighted the benefits of SPG in promoting overall joint health and alleviating OA symptoms, its precise molecular mechanisms remain to be fully elucidated. To address this gap, the present study investigated the therapeutic efficacy of SPG in a monosodium iodoacetate (MIA)-induced OA mouse model. Specifically, we focused on its capacity to mitigate joint pain and modulate OA progression by evaluating the regulatory balance between anabolic and catabolic pathways, as well as the associated inflammatory processes.

## 2. Materials and Methods

### 2.1. Salmon Nasal Cartilage Proteoglycan (SPG) Source

The SPG used throughout this study, derived from salmon nasal cartilage, was obtained as a spray-dried powder extract (Lot 232540) from Ichimaru Pharcos, Co., Ltd. (Gifu, Japan). Comprehensive details regarding the preparation and characteristics of this specific extract have been previously documented [43].

### 2.2. Animal Care and Ethics Approval

Six-week-old male C57BL/6J mice, initially weighing between 21 and 23 g, were purchased from Daehan Bio Link Co., Ltd. (Eumseong, Chungbuk, Republic of Korea). All animals were housed within the dedicated animal facility located at the College of Pharmacy, Chungbuk National University (Cheongju, Chungbuk, Republic of Korea). Prior to experimentation, the animals underwent a seven-day adaptation period in a standard environment maintained at 23 ± 2 °C, under a controlled 12-h light/dark cycle. Throughout the study, the mice were given unrestricted access to filtered tap water and a standard rodent chow diet (Rodfeed^®^, Daehan Bio Link). Every animal protocol strictly conformed to the Korean National Animal Welfare Guide and the corresponding National Animal Welfare Legislation. The entire set of experimental procedures was formally reviewed and approved by the Institutional Animal Care and Use Committee (IACUC) at Chungbuk National University (Approval Number CBNUA-2271-24-01, 29 March 2024).

### 2.3. Experimental Design and Administration

A total of 180 mice were incorporated into this research endeavor. The requisite sample size (*n* = 10–12 per group) was determined by adhering to established methodological standards for the assessment of pain and cartilage integrity within the monosodium iodoacetate (MIA)-induced OA model [46]. This size was strategically selected to guarantee sufficient power for detecting biologically meaningful differences and maintaining consistency with previous findings, while simultaneously respecting ethical directives for animal use and minimizing the number of animals required. To mitigate selection bias and ensure group comparability, subjects were assigned using stratified randomization based on their initial body weight, thereby achieving equivalent mean body weight across the experimental groups at baseline. The randomization sequence itself was generated via a computer-based pseudo-random sequence generator.

**Figure 1:**
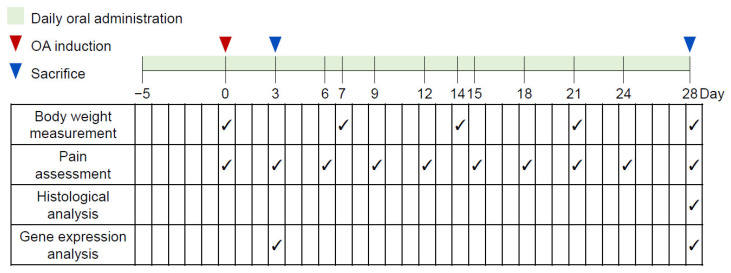
Graphical summary of the experimental protocol and schedule. The provided diagram illustrates the complete study duration and the segmentation into three distinct, independent cohorts. Mice (*n* = 10–12 per group) received daily treatments via oral gavage (normal saline, SPG, or CLX). This dosing regimen commenced 5 days before OA induction (designated as Day 0) and continued until the specific endpoint for each cohort. Animal body weights were recorded at the beginning of every week. Articular pain levels were evaluated every three days until Day 24, with the final measurement taken on Day 28. Subjects were euthanized at two distinct timepoints (Day 3 or Day 28) for subsequent sample harvesting. Specifically, knee joint tissues were collected from 6 to 10 animals per group for histological analysis (at Day 28), and articular cartilage was collected from 6 to 12 animals per group for mRNA expression analysis (at Day 3 or Day 28), depending on the experimental cohort. Arrowheads (√) represent the time points of experimental procedures. OA, osteoarthritis; SPG, salmon nasal cartilage proteoglycan; CLX, celecoxib.

The experimental subjects were subsequently divided into three independent cohorts corresponding to distinct analytical endpoints: (1) functional assessment (pain), histology, and immunohistochemistry; (2) mRNA expression analysis for early-stage OA (Day 3); and (3) mRNA expression analysis for the late-stage OA (Day 28). Each of these cohorts was structured into five distinct experimental groups (*n* = 10–12 per group): a normal control group that received an intra-articular injection of normal saline (Sham), a disease model control group induced by MIA injection (Control), a positive comparator receiving celecoxib (CLX, 20 mg/kg/day), and two separate groups received escalating doses of SPG. The applied SPG dosages for mice (50 or 100 mg/kg/day) were precisely derived from the human equivalent daily dosages of 250 and 500 mg, respectively, by implementing established interspecies dose conversion guidelines [47]. All animals received daily oral treatment via gavage. Treatment commenced 5 days prior to OA induction and continued post-induction until the designated experimental termination time point (either 3 or 28 days). A corresponding volume of vehicle (normal saline) was administered to both the Sham and Control groups. The comprehensive details regarding the group composition and overall experimental timeline are presented in Table 1 and Figure 1.

Animal welfare and general health status were closely monitored for throughout the experiment. At the conclusion of each designed experimental time point, subjects were euthanized using CO_2_ inhalation for the subsequent harvest of tissues or blood samples.

### 2.4. Induction of Osteoarthritis Using Monosodium Iodoacetate (MIA)

OA was established in the animals following a previously validated protocol [46]. Initially, the subjects underwent inhalation anesthesia using 2.0–3.5% isoflurane (Piramal Critical Care, Bethlehem, PA, USA). The OA condition was subsequently induced by an intra-articular injection of MIA solution (Cat No. 57858-5G-F, Sigma-Aldrich, St. Louis, MI, USA) at a dosage of 1 mg/10 μL into the left hind limb knee joint, positioning the injection site inferior to the patella. Conversely, animals in the Sham and Control groups were injected with an equivalent volume of normal saline. Post-injection, the mice were closely observed until their complete recovery from the anesthetic effects before being returned to their designated cages. Criteria for exclusion included failure to recover from the OA induction procedure or the presence of significant general health issues. They were maintained on their established daily oral treatment (SPG, CLX, or vehicle) until the predefined time point for experimental termination.

### 2.5. Body Weight Measurement

For general health monitoring throughout the experimental duration, animal body weights were precisely recorded at 7-day intervals using a weighing scale (SPX2202KR, OHAUS Corporation, Parsippany, NJ, USA).

### 2.6. Pain Assessment (Nociception Measurement)

Articular pain severity was quantitatively determined by measuring the differential weight distribution across the ipsilateral (OA-affected) and contralateral (unaffected) posterior limbs. This assessment utilized an incapacitance meter (Model 600MR, IITC Life Science, Inc., Woodland Hills, CA, USA), following methodologies established in prior reports [46,48]. For each testing session, the animals were cautiously positioned within a specialized, angled acrylic container. The sensors located centrally beneath the animals were used to record the force exerted by the ipsilateral (left) and the contralateral (right) hind paws separately. Each individual measurement was registered over a period of 5 s. The analytical value was subsequently computed as the mean of three consecutive readings. These raw force measurements were then converted to a percentage indicating the weight distribution, calculated as the ipsilateral weight divided by the sum of the weights recorded by both hind limbs, as shown in the equation below.

$$\mathrm{Weight}\;\mathrm{bearing}\;(\%)=\frac{\mathrm{Ipsilateral weight}}{(\mathrm{Ipsilateral weight}+\mathrm{Contralateral}\;\mathrm{weight})}\;\times 100$$

### 2.7. Histopathological Evaluation

Twenty-eight days following OA induction, the left hind limbs were harvested and prepared for subsequent histological examination. The specimens were initially fixed for 24 h at 4 °C in 4% paraformaldehyde solution. Decalcification then proceeded for two weeks, utilizing 5% formic acid. Following this step, the tissues underwent a sequential dehydration process using an ascending ethanol series before being embedded in paraffin blocks. Sectioning was performed on the resulting blocks using a microtome (Model CM 1850, Leica Microsystems, Wetzlar, Germany) to yield 5 μm thick sagittal slices.

To evaluate the structural integrity of the cartilage, the prepared sections were stained with hematoxylin and eosin (H&E) solution (Leica Microsystems) and the Safranin-O/Fast Green Staining kit (IHC World, Wookstock, MD, USA). After mounting the slides with Permount mounting medium (Fisher Scientific International, Inc., Pittsburgh, PA, USA), images were digitally captured using a light microscope (Model DM2500 LED, Leica Microsystems) at both 5× and 20× magnifications.

All stained slides were subjected to a double-blind, independent evaluation [49,50,51]. Cartilage damage was quantified using the established Mankin scoring system (ranging from 0 to 13), and the extent of synovitis was assessed using Krenn’s synovitis scoring system (ranging from 0 to 6).

### 2.8. Immunohistochemistry

To determine the protein expression patterns of essential cartilage matrix components within the joint tissue, an immunohistochemistry protocol was executed on the paraffin-embedded sections. Initially, the sections were processed for deparaffinization and subsequent rehydration. Antigen retrieval was achieved by incubating the sections overnight in a 10 mM sodium citrate buffer (pH 6.0) at 60 °C. Following rinses with phosphate-buffered saline (PBS), non-specific binding was blocked by incubation with 5% normal goat serum in PBS. The immunostaining sequence continued with an overnight incubation 4 °C utilizing the following primary antibodies: anti-collagen type II alpha 1 (COL2A1, SantaCruz Biotechnology, Dallas, TX, USA; Cat No. sc-52658, 1:50); and anti-cartilage oligomeric protein (COMP, Abcam, Waltham, MA, USA; Cat No. ab231977, 1:100). After thorough washing to remove unbound primary antibody, the sections were incubated with the biotinylated secondary antibodies for 2 h at ambient temperature. Visualization of the Immunocomplexes was performed using the 3,3’-diaminobenzideine (DAB) detection kit (Vector Laboratory, Newark, CA, USA), strictly following the manufacturer’s instructions. The finalized, stained slides were imaged under a light microscope (Model DM2500 LED, Leica Microsystems) at a magnification of 5× and 20×.

### 2.9. Molecular Quantification via Real-Time PCR

The determination of gene expression profiles commenced with the isolation of total RNA from the mouse articular cartilage tissues harvested at either Day 3 or Day 28 post-OA induction. This extraction procedure utilized the Total RNA isolation kit (Hybrid—R^TM^, GeneAll Biotechnology, Seoul, Republic of Korea), with strict adherence to the manufacturer’s instructions. The resulting purified RNA was subsequently reverse-transcribed into complementary DNA (cDNA) using the High-Capacity cDNA Reverse Transcription Kit (Applied Biosystems, Foster City, CA, USA). Following cDNA synthesis, quantitative real-time PCR (RT-qPCR) assays were executed using a qPCR master mix (THUNDERBIRDTM SYBR^®^, Toyobo, Osaka, Japan) and pre-designed, custom oligonucleotide primers. β-actin served as the internal reference control for normalization. All RT-qPCR reactions were performed using as Real-Time PCR system (QuantStudio 3, Thermo Fisher Scientific, Wal tham, MA, USA). The specific nucleotide sequences employed for the primers are fully detailed in Table 2. Relative quantification of gene expression was determined through the application of the 2^−ΔΔCT^ method.

### 2.10. Statistical Analysis

All numerical data collected are uniformly expressed as the mean ± standard error of the mean (SEM). The sample size, denoted by ‘*n*’ (number of animals or replicates), is precisely noted for each dataset. Statistical significance testing was executed using different methods, conditional on the nature of the comparison. Statistical significance was evaluated using one-way analysis of variance (ANOVA), followed by Dunnett’s post-hoc test to determine differences between the Control group and the other experimental groups (Sham, CLX 20 mg/kg, SPG 50 mg/kg, and SPG 100 mg/kg). For data involving multiple time points, repeated-measure (RM) two-way ANOVA was performed, followed by Dunnett’s post hoc test for multiple comparisons. All computations were performed using GraphPad Prism software (Version 8.4.2, GraphPad Software, Boston, MA, USA). A probability (*p*) value set below 0.05 (*p* < 0.05) was deemed statistically significant.

## 3. Results

### 3.1. SPG Does Not Alter Body Weight in MIA-Induced OA Mice

We initially assessed animal body weight changes to verify any potential systemic effect of MIA or the test substance treatment. As shown in Supplementary Figures S1 and S2, all MIA-treated groups, with the exception of the SPG 100 mg/kg group, experienced a transient body weight reduction compared to the Sham group on Day 7 post-OA induction. Although the CLX (Days 21 and 28) and Control (Day 21) groups showed temporary weight differences relative to the Sham group later in the experiment, all MIA-treated groups consistently recovered their body weight after Day 14. This consistent recovery trend indicates that the overall health status of the animals remained stable throughout the experimental period, indicating that SPG administration did not induce significant systemic health complications.

### 3.2. SPG Exhibits Significant Alleviation of Joint Pain in MIA-Induced OA Mice

**Figure 2:**
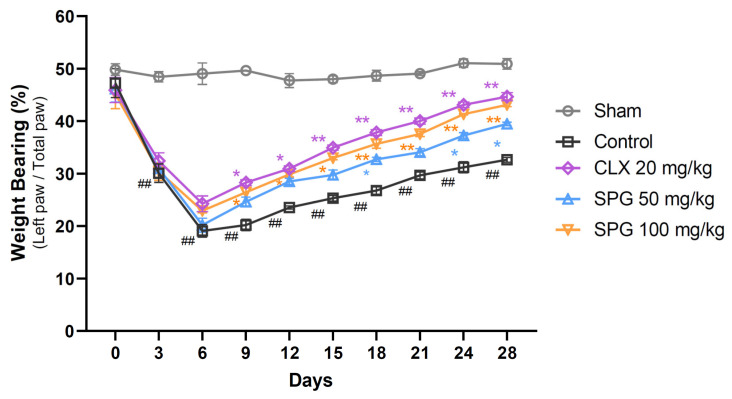
Effect of SPG treatment on joint pain in MIA-induced OA mice. The distribution of weight supported by the hind limbs was quantified as the primary indicator of joint pain in all experimental subjects (*n* = 10–12 per group). Measurements were consistently logged every three days until Day 21, culminating in the final assessment on Day 28. Data are presented as the calculated percentage of load borne by the ipsilateral (OA-affected) paw relative to the total force supported by both posterior limbs. Values are depicted as the mean ± SEM. Statistical significance was ascertained using repeated-measure two-way ANOVA followed by Dunnett’s post hoc test. The legend for significant differences is as follows: ## *p* < 0.01 compared to the Sham group; * *p* < 0.05 compared to the Control group; and ** *p* < 0.01 compared to the Control group. MIA, monosodium iodoacetate; OA, osteoarthritis; SPG, salmon nasal cartilage proteoglycan; CLX, celecoxib.

The analgesic activity of orally delivered SPG was assessed in the MIA-induced OA model by measuring the differential weight distribution of the posterior limbs (Figure 2). Quantification of nociception confirmed that the Sham group maintained an equitable load distribution throughout the observation period. In sharp contrast, the Control group exhibited a marked, persistent reduction in the weight supported by the ipsilateral (OA-affected) hind limb compared to the Sham animals over the entire study duration. Significantly, all active treatment groups (CLX 20 mg/kg, SPG 50 mg/kg, and SPG 100 mg/kg) displayed enhanced weight-bearing capability when compared to the Control group. Specifically, the high-dose SPG group (100 mg/kg) demonstrated significant recovery from Day 9 post-OA induction, reaching an efficacy level comparable to that of the positive comparator, CLX. These findings strongly indicate that the oral administration of SPG is effective in reducing joint pain in mice challenged with MIA-induced OA.

### 3.3. SPG Attenuates Cartilage Degeneration in MIA-Induced OA

**Figure 3:**
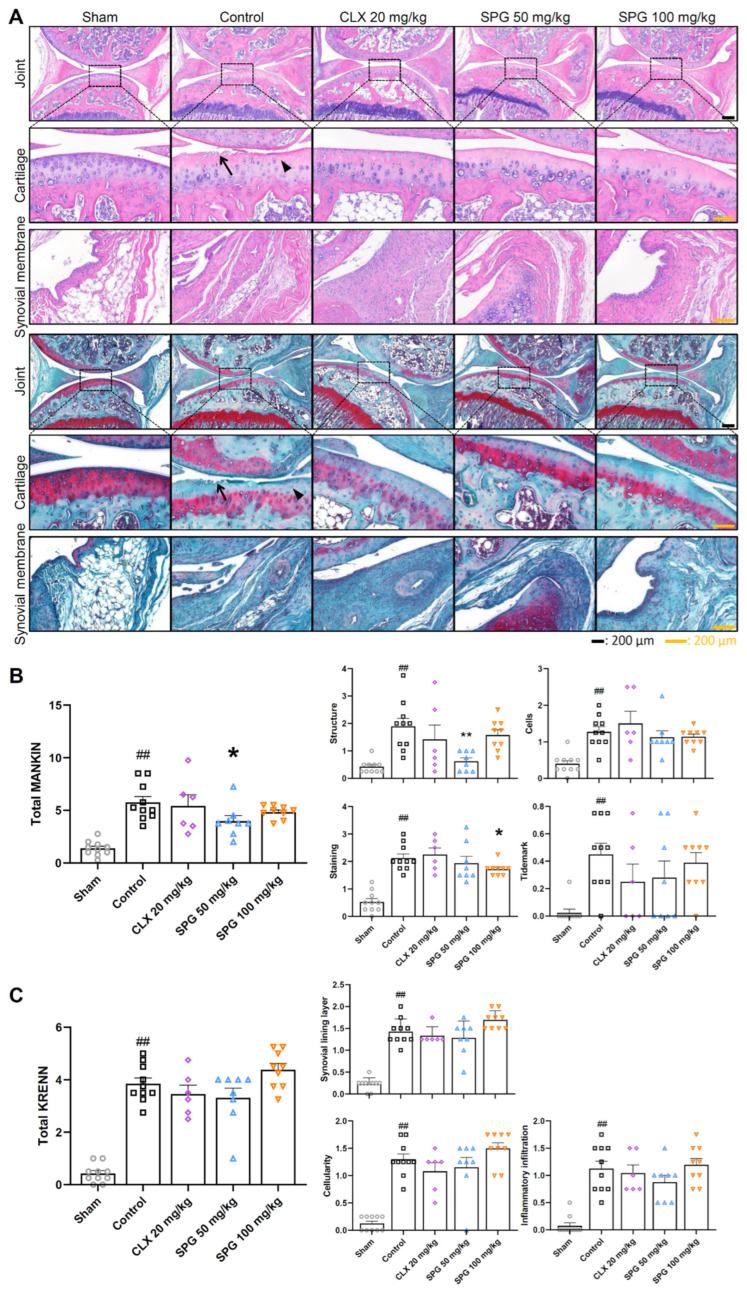
Histomorphological assessment of SPG’s chondroprotective and anti-synovitis efficacy in MIA-induced OA mice: (**A**) Representative micrographs of knee joint cartilage and synovium. Structural morphology of the cartilage was evaluated using hematoxylin and eosin (H&E) staining (upper panels), while the content of proteoglycan was analyzed via Safranin-O/Fast Green staining (lower panels). Arrows indicate cartilage surface irregularities, and arrowheads indicate chondrocyte loss. Images, which are representative of each experimental group were captured using a light microscope at 5× and 20× optical magnification. (**B**) Cartilage integrity was quantitatively assessed by grading the lesion severity based on the modified Mankin scoring system, yielding a total Mankin score. (**C**) Synovitis and synovial fibrosis were quantified by grading the extent of synovial membrane lesions using the established Krenn’s synovitis scoring system, resulting in a total Krenn’s score. Symbols represent the following experimental groups: hollow circles (Sham), squares (Control), rhombuses (CLX 20 mg/kg), triangles (SPG 50 mg/kg), and inverted triangles (SPG 100 mg/kg). Data are presented as mean ± SEM (*n* = 6–10 per group). Statistical significance was ascertained using one-way ANOVA, followed by Dunnett’s post hoc test. The legend for significant differences is as follows: ## *p* < 0.01 compared to the Sham group; and * *p* < 0.05 compared to the Control group, ** *p* < 0.01 compared to the Control group. MIA, monosodium iodoacetate; OA, osteoarthritis; SPG, salmon nasal cartilage proteoglycan; CLX, celecoxib.

To evaluate the protective effects of oral SPG administration on cartilage degeneration, we performed histological assessments of joint articular cartilage and synovium using H&E staining (for overall morphology) and Safranin-O/Fast Green (to assess proteoglycan content), as illustrated in Figure 3A. These findings were quantified using the Mankin score (for cartilage damage) and Krenn’s synovitis score (for synovitis and synovial fibrosis).

The Sham group displayed normal cartilage characteristics, including a smooth articular surface and orderly chondrocyte arrangement, alongside typical synovial morphology. In stark contrast, the Control group exhibited severe OA pathology, characterized by marked cartilage surface irregularities, substantial structural destruction in the superficial and middle zones, pronounced chondrocyte loss, and notable synovial hypertrophy. These findings were strongly corroborated by significantly elevated scores in both the Mankin score and Krenn’s synovitis score in the Control group compared to the Sham group.

Both CLX and SPG administration resulted in a substantial amelioration of cartilage destruction in the H&E sections (Figure 3A, upper panels). Furthermore, Safranin-O/Fast Green staining demonstrated a dose-dependent enhancement of Safranin-O intensity in the SPG-treated groups, particularly SPG 100 mg/kg, compared to the Control group, signifying improved proteoglycan preservation (Figure 3A, lower panels). Quantitatively, the Mankin scoring system (Figure 3B) confirmed a reduction in the total score for both the SPG 50 mg/kg and SPG 100 mg/kg groups compared to the Control group. Notably, the SPG 50 mg/kg group showed a statistically significant improvement, specifically in the Structure category. However, no significant statistical difference was detected between the SPG-treated groups and the Control group concerning synovial appearance or Krenn’s synovitis scoring system scores (Figure 3C). Overall, these results confirm that oral SPG administration effectively inhibits cartilage degeneration and preserves proteoglycan content in MIA-induced OA mice.

### 3.4. SPG Selectively Modulates Pro-Inflammatory Cytokine Gene Expression by Reducing IL-6 at the Late Stage of MIA-Induced OA

**Figure 4:**
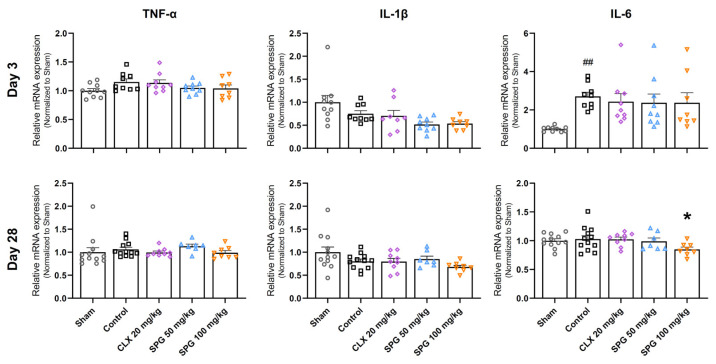
Effect of oral SPG administration on pro-inflammatory cytokine gene expression within articular cartilage of MIA-induced OA mice. The relative mRNA expression of key pro-inflammatory cytokines, including tumor necrosis factor-alpha (TNF-α), interleukin-1 beta (IL-1β), and interleukin-6 (IL-6), was quantified in articular cartilage samples via RT-qPCR. Results are categorized by two distinct temporal assessments: Day 3 (upper panels), which represents the initial phase of OA development, and Day 28 (lower panels), which corresponds to the late-stage disease. Normalization of data was conducted against β-actin expression levels. Symbols represent the following experimental groups: hollow circles (Sham), squares (Control), rhombuses (CLX 20 mg/kg), triangles (SPG 50 mg/kg), and inverted triangles (SPG 100 mg/kg). Data are presented as the mean ± SEM (*n* = 7–12 per group). Differences between groups were determined utilizing one-way ANOVA, followed by Dunnett’s post hoc test. The legend for significant differences is as follows: ## *p* < 0.01 compared to the Sham group; * *p* < 0.05 compared to the Control group. MIA, monosodium iodoacetate; OA, osteoarthritis; SPG, salmon nasal cartilage proteoglycan; CLX, celecoxib.

To clarify the molecular mechanism behind the chondroprotective effects observed from oral SPG administration, we employed RT-qPCR to measure the expression of major pro-inflammatory cytokines, specifically TNF-α, IL-1β, and IL-6, within the articular cartilage (Figure 4). The inflammatory status was relatively quiescent at Day 3 post-OA induction, which marks the early phase of the disease. During this early phase, only IL-6 mRNA expression was found to be significantly elevated in the Control group compared to the Sham group. Furthermore, no significant alterations were detected in the transcription levels of TNF-α or IL-1β at this specific time point. Although a numerical trend showing reduced IL-6 expression was observed across all three active treatment arms (CLX 20 mg/kg, SPG 50 mg/kg, SPG 100 mg/kg), these differences did not achieve statistical significance relative to the Control group. By Day 28, representing the advanced OA stage, the overall inflammatory profile appeared to subside across all groups, with no significant differences identifiable for most markers examined. Notwithstanding this general subsidence, the high-dose SPG group (SPG 100 mg/kg) specifically exhibited a statistically significant attenuation of IL-6 mRNA expression when contrasted with the Control group. In summation, these outcomes suggest that the ability of oral SPG administration to broadly suppress the expression of pro-inflammatory cytokines within the cartilage is limited. The singular, notable exception was the specific and significant IL-6 gene reduction documented at the high-dose of SPG during the late stage of OA progression.

### 3.5. SPG Sustains Cartilage Matrix Integrity and Attenuates Homeostatic Anabolic Upregulation at the Late Stage of MIA-Induced OA

**Figure 5:**
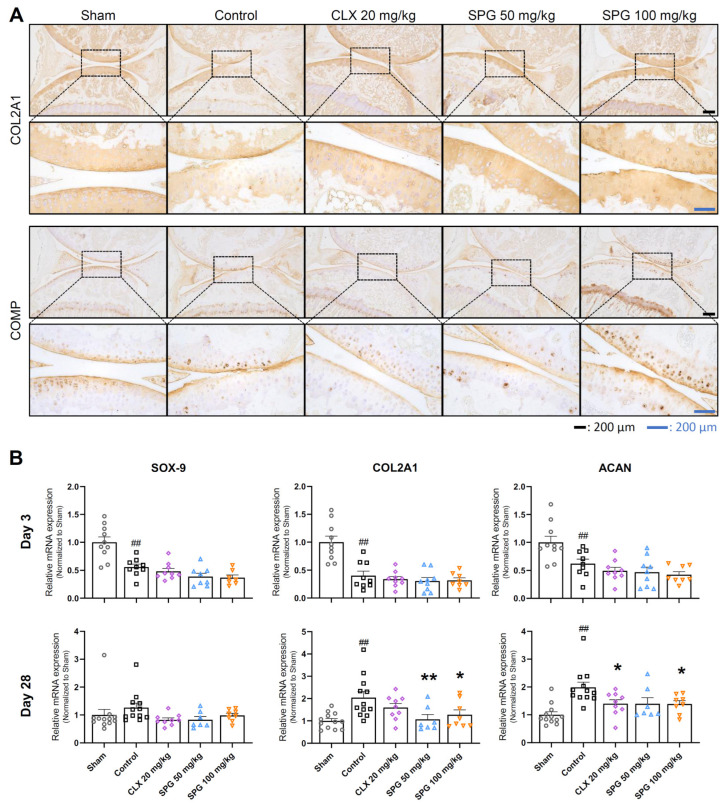
Effect of oral SPG administration on cartilage matrix constituents and the transcription factor governing synthesis in MIA-induced OA mice: (**A**) Immunohistochemistry was performed to evaluate the content of essential cartilage components. The results display the staining for collagen type II alpha 1 chain (COL2A1) (upper panels) and anti-cartilage oligomeric protein (COMP) (lower panels). Micrographs, which are representative of each experimental group (*n* = 3 per group), were captured under a light microscope at 5× and 20× optical magnification. (**B**) The relative mRNA expression of the anabolic transcription factor SOX-9 and the principal matrix components COL2A1, and aggrecan (ACAN) was quantified via RT-qPCR. Data were normalized relative to β-actin expression levels. Symbols represent the following experimental groups: hollow circles (Sham), squares (Control), rhombuses (CLX 20 mg/kg), triangles (SPG 50 mg/kg), and inverted triangles (SPG 100 mg/kg). Data are presented as mean ± SEM (*n* = 7–12 per group). Differences between groups were determined utilizing one-way ANOVA, followed by Dunnett’s post hoc test. The legend for significant differences is as follows: ## *p* < 0.01 compared to the Sham group; * *p* < 0.05 compared to the Control group; and ** *p* < 0.01 compared to the Control group. MIA, monosodium iodoacetate; OA, osteoarthritis; SPG, salmon nasal cartilage proteoglycan; CLX, celecoxib.

To determine the extent of long-term cartilage component preservation by oral SPG administration, we utilized both immunohistochemistry (IHC) and RT-qPCR. IHC was performed at the Day 28 endpoint to assess the protein content of the major cartilage matrix elements: COL2A1, a primary constituent of type II collagen, and COMP, vital for stabilizing the collagen networks, as visualized in Figure 5A. Concurrently, we quantified the mRNA expression of the master anabolic transcription factor SOX-9, along with the matrix components COL2A1 and aggrecan (ACAN), at both the early (Day 3) and late (Day 28) time points (Figure 5B).

Analysis of protein expression at Day 28 revealed a stark contrast between the healthy and pathological states. While the COL2A1 protein was ubiquitously present across all cartilage zones in the Sham group, it was severely diminished or absent in the compromised superficial and middle layers of the Control group. Favorably, treatment with both CLX and SPG doses resulted in well-maintained COL2A1 expression throughout the entire cartilage depth (Figure 5A, upper panels). Regarding the COMP protein, the Control group demonstrated enhanced expression in the middle and deep zones, potentially signifying a vigorous compensatory repair mechanism. The SPG-treated animals showed a similar, or in some cases more pronounced, COMP expression profile than the Control group, pointing toward the high protective action of SPG (Figure 5A, lower panels).

At the Day 3 early stage, the transcriptional activity of all three anabolic markers, SOX-9, COL2A1, and ACAN, was markedly suppressed in the Control group compared to the Sham group, indicating initial chondrocyte dysfunction (Figure 5B, upper panels). By Day 28, this expression pattern was dramatically reversed. At this late stage, the Control group showed significantly elevated mRNA expression levels of SOX-9, COL2A1, and ACAN compared to the Sham group. This increase likely reflects a robust, albeit ultimately inadequate, compensatory anabolic response to severe matrix loss. In striking contrast, the CLX- and SPG-treated groups showed an attenuated need for such homeostatic upregulation, maintaining markedly lower mRNA levels of these markers than the Control group. Specifically, COL2A1 transcription was significantly lower in both the SPG 50 and 100 mg/kg groups. Additionally, ACAN expression was significantly suppressed in the CLX 20 mg/kg and SPG 100 mg/kg groups compared to the Control group (Figure 5B, lower panels).

Collectively, the integration of protein and gene expression data strongly suggests that oral SPG administration preserves the structural integrity of key cartilage components. By preventing extensive matrix degradation, SPG effectively attenuates the chronic activation of compensatory anabolic pathways typically observed during the late stages of MIA-induced OA.

### 3.6. SPG Mitigates Cartilage-Degrading Enzyme Expression and Modifies Their Endogenous Inhibitors in MIA-Induced OA

We investigated the influence of oral SPG treatment on the gene expression of matrix-degrading enzymes, specifically matrix metalloproteinases (MMPs) and a disintegrin and metalloproteinase with thrombospondin motif (ADAMTS) family members, recognized as central mediators of cartilage destruction, at both Day 3 (the early phase) and Day 28 (the late phase). Concurrently, the expression of tissue inhibitors of metalloproteinases (TIMPs), the natural endogenous inhibitors of these enzymes, was also assessed.

**Figure 6:**
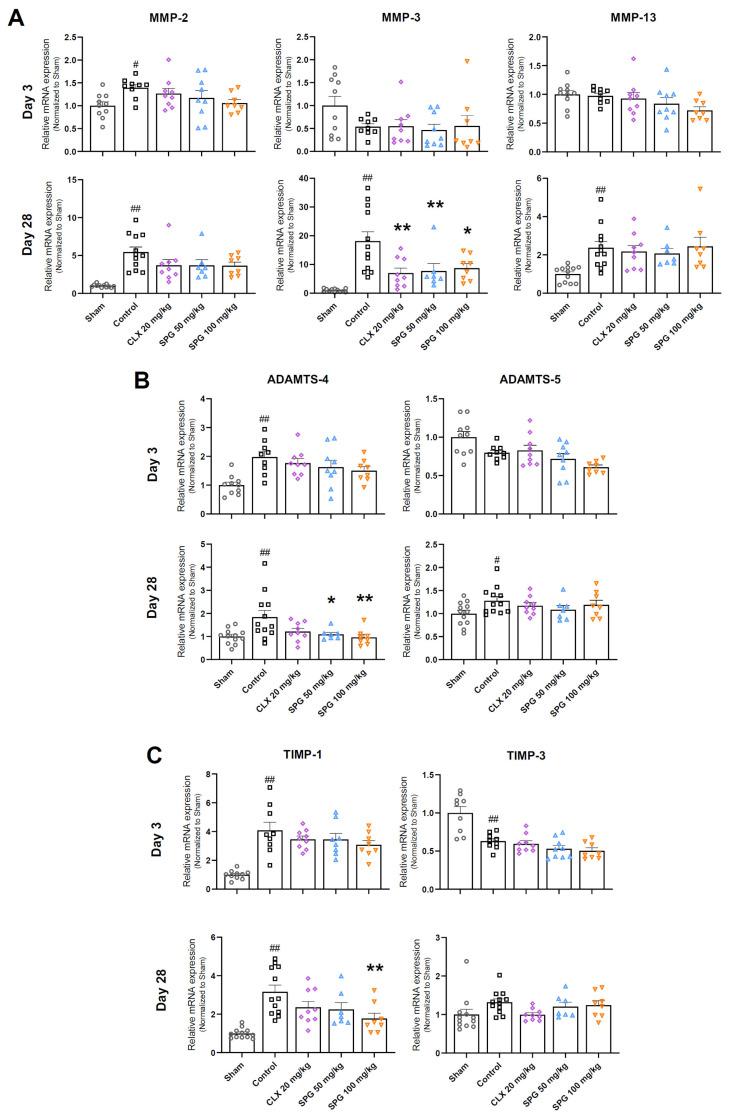
Effect of oral SPG administration on cartilage-degrading enzymes and their endogenous inhibitors’ gene expression in MIA-induced OA mice. The mRNA expression of cartilage-degrading enzymes and their endogenous inhibitors was quantified by RT-qPCR in mouse cartilage tissue samples collected on Day 3 (upper panels) and Day 28 (lower panels) post-OA induction: (**A**) matrix metalloproteinases (MMPs): MMP-2, MMP-3, and MMP-13. (**B**) Aggrecanases (ADAMTS): ADAMTS-4 and ADAMTS-5. (**C**) Endogenous inhibitors (TIMPs): TIMP-1 and TIMP-3. Data were normalized relative to β-actin expression levels. Symbols represent the following experimental groups: hollow circles (Sham), squares (Control), rhombuses (CLX 20 mg/kg), triangles (SPG 50 mg/kg), and inverted triangles (SPG 100 mg/kg). Data are presented as mean ± SEM (*n* = 7–12 per group). Differences between groups were determined utilizing one-way ANOVA, followed by Dunnett’s post hoc test. The legend for significant differences is as follows: # *p* < 0.05 compared to the Sham group; ## *p* < 0.01 compared to the Sham group; * *p* < 0.05 compared to the Control group; and ** *p* < 0.01 compared to the Control group. MIA, monosodium iodoacetate; OA, osteoarthritis; SPG, salmon nasal cartilage proteoglycan; CLX, celecoxib. MMP-2, matrix metalloproteinase-2; MMP-3, matrix metalloproteinase-3; MMP-13, matrix metalloproteinase-13; ADAMTS-4, A disintegrin and metalloproteinase with thrombospondin motifs-4; ADAMTS-5, A disintegrin and metalloproteinase with thrombospondin motifs-5; TIMP-1, tissue inhibitor of metalloproteinase-1; TIMP-3, tissue inhibitor of metalloproteinase-3.

At day 3 post-OA induction, only MMP-2 levels were significantly elevated in the Control group compared to the Sham group. Although the SPG-treated groups exhibited a downward trend in MMP-2 expression, the difference did not reach statistical significance (Figure 6A, left upper panel). Conversely, MMP-3 gene expression was lower in the Control, CLX, and SPG-treated groups relative to the Sham group, though these changes were not statistically significant (Figure 6A, middle upper panel). Regarding MMP-13, a slight attenuation was observed in the SPG 100 mg/kg group compared to the Control group, but this remained non-significant (Figure 6A, right upper panel). ADAMTS-4 expression increased markedly in the Control group relative to the Sham group; however, the reducing tendencies in the CLX- and SPG-treated groups did not achieve statistical significance (Figure 6B, left upper panel). Finally, ADAMTS-5 gene expression was diminished across all experimental groups compared to the Sham group (Figure 6B, right upper panel). By day 28 post-OA induction, the changes in enzyme expression became more pronounced. The transcription of MMP-2, MMP-3, MMP-13, ADAMTS-4, and ADAMTS-5 was significantly upregulated in the Control group relative to the Sham group. Notably, treatment with CLX and both doses of SPG (50 and 100 mg/kg) reduced the mRNA levels of MMP-2, MMP-3, and ADAMTS-4 compared to the Control group. Specifically, MMP-3 expression was significantly downregulated across all treatment groups. Furthermore, both the SPG 50 and 100 mg/kg groups showed a significant decrease in ADAMTS-4 levels relative to the Control group. In contrast, no statistically significant differences were observed in MMP-13 and ADAMTS-5 expression between the Control group and the treated groups at this stage (Figure 6).

Regarding the endogenous inhibitors, early-stage (Day 3) TIMP-1 expression was significantly elevated in the Control group compared to the Sham group, whereas the CLX and SPG groups showed a non-significant downward trend (Figure 6C, left upper panel). At the same time point, TIMP-3 transcription was lower across the Control, CLX, and SPG groups relative to the Sham group, although these differences did not reach statistical significance (Figure 6C, right upper panel). By the late stage (Day 28), TIMP-1 expression remained significantly higher in the Control group than in the Sham group. However, CLX and SPG treatments attenuated this increase, with the SPG 100 mg/kg group exhibiting a statistically significant reduction compared to the Control group (Figure 6C, left lower panel). Conversely, TIMP-3 expression in the Control group showed a non-significant upward trend compared to the Sham group, a pattern that was not observed in the CLX- or SPG-treated groups (Figure 6C, right lower panel).

Viewed collectively, these results suggest that oral SPG administration effectively curtails matrix breakdown by attenuating the expression of cartilage-degrading enzymes and modulating the transcription of their endogenous inhibitors. These effects are most prominent during the chronic phase of OA, suggesting that SPG preserves cartilage integrity by rebalancing articular homeostasis.

## 4. Discussion

OA is a highly pervasive and debilitating joint disorder characterized by a triad of pathologies: chronic pain, persistent low-grade inflammation, and the gradual degradation of the articular cartilage [52]. In this study, we utilized the MIA-induced OA mouse model, which faithfully recapitulates the structural, biochemical, and functional characteristics of human OA, to evaluate the therapeutic potential of oral SPG administration. Our findings demonstrate that SPG provides a dual benefit of functional pain mitigation (analgesic effects) and structural chondroprotection by restoring the delicate metabolic balance within the joint environment.

Articular pain represents the dominant clinical symptom of OA, typically exacerbated by mechanical loading and bearing of weight, thereby resulting in substantial impairment [53]. The MIA challenge specifically induces nociception through initial chemical irritation followed by secondary tissue destruction, leading to both peripheral and central sensitization. Our results established that oral SPG administration significantly improved weight-bearing distribution, a key indicator of pain relief, with efficacy comparable to the positive control (Figure 2). This analgesic outcome suggests that SPG, or its bioactive components, can modulate nociceptive signaling. More importantly, the prolonged pain relief observed in the late stage indicates that SPG may alleviate pain indirectly by attenuating the underlying joint damage that acts as a mechanical trigger for joint discomfort.

The inflammatory process is recognized as an essential contributor to OA pathogenesis, characterized by the local release of pro-inflammatory signaling molecules such as TNF-α, IL-1β, and IL-6 originating from the synovial tissue and activated chondrocytes [54,55]. These mediators subsequently establish a catabolic milieu, which ultimately jeopardizes the structural integrity of the cartilage matrix [56,57]. Although some marine proteoglycans are known for broad-spectrum anti-inflammatory activity [58], SPG appears to function as a more targeted modulator. Our findings indicated that SPG significantly and specifically downregulated IL-6 expression during the chronic phase (Day 28), particularly at the high dose of 100 mg/kg, whereas it showed limited impact on the broader suppression of TNF-α and IL-1β. This selective action is particularly important, as IL-6 is a key mediator linking mechanical stress, inflammation, and catabolism, often driving the chronic phase of OA. This suggests that SPG may not function as a broad-spectrum anti-inflammatory agent, but rather as a selective modulator, targeting the chronic, late-stage signaling pathway involving IL-6 at sufficient concentrations.

The chondroprotective efficacy of SPG was further validated by the maintenance of cartilage matrix structure and lower Mankin scores (Figure 3). The loss of articular tissue is primarily driven by an increase in matrix-degrading enzymes like MMPs and ADAMTS, which target type II collagen and aggrecan. Our findings show that SPG counters this process by downregulating MMP-3 and ADAMTS-4 while simultaneously stabilizing their natural inhibitors: TIMP-1 and TIMP-3 (Figure 6). This observation is consistent with prior research documenting the anti-catabolic function of SPG against MMP-3 and MMP-13 in various OA models [58]. By shifting the proteolytic balance toward inhibition, SPG effectively safeguards the extracellular matrix from irreversible destruction. This mechanism, based on regulating the enzyme-to-inhibitor ratio, represents a classical and highly efficacious strategy for joint preservation.

Perhaps the most significant finding is SPG’s role in regulating the abnormal anabolic response. During advanced OA, chondrocytes often increase matrix synthesis as a desperate compensatory measure for rapid degradation [17,59]. This drive was clearly evidenced in the Control group at Day 28, which showed an elevation in COMP protein expression and a significant increase in the gene expression of SOX-9, COL2A1, and ACAN (Figure 5). This robust, yet ultimately failing, attempt represents a chronic compensatory anabolic signaling burden. Crucially, the SPG administration groups, despite exhibiting high protective activity, preserved COL2A1 and increased COMP integrity (Figure 5A), showed significantly reduced gene expression of these anabolic markers compared to the Control group at Day 28. This implies that by effectively mitigating initial catabolic damage, SPG treatment reduced the necessity for the chronic and energetically demanding compensatory response. This rebalancing of the ECM homeostasis, rather than merely stimulating synthesis, represents a sophisticated chondroprotective mechanism.

The findings of this study have significant clinical and nutritional implications. From a clinical perspective, SPG demonstrates potential as a complementary therapeutic agent for managing OA by effectively mitigating joint pain and preserving cartilage integrity through the regulation of catabolic pathways. Its ability to restore the metabolic balance in joint tissues suggests it could reduce reliance on conventional NSAIDs, which often carry gastrointestinal side effects. From a nutritional standpoint, as a naturally derived compound from salmon, SPG offers a safe and sustainable functional food ingredient. Incorporating SPG into dietary strategies may provide a preventive approach for aging populations or individuals at high risk of joint degeneration, supporting overall mobility and quality of life.

Despite these significant findings, several limitations of this study warrant consideration. First, while we established the regulatory effect of SPG on matrix metabolism, the precise upstream signaling pathways and molecular targets remain to be fully characterized. Second, this study utilized only male mice to ensure group homogeneity; consequently, potential sex-dependent differences in SPG efficacy should be investigated in future research. Finally, although the MIA-induced OA model effectively reflects OA pathology, it cannot fully replicate the complex, chronic progression of age-related OA in humans. Therefore, subsequent long-term clinical investigations are imperative to verify whether the chondroprotective and analgesic effects observed in this animal model translate effectively to human subjects in terms of safety and efficacy.

## 5. Conclusions

In conclusion, this investigation offers strong evidence supporting the therapeutic viability of orally administered SPG as a promising intervention for OA. Our findings derived from the MIA-induced OA murine model clearly demonstrate that SPG treatment was efficacious in not only providing robust pain relief but also ensuring significant protection of cartilage integrity against degradation. At the mechanistic level, we identified that SPG markedly repressed the transcription of major cartilage-degrading enzymes, notably MMPs and ADAMTS, particularly during the advanced phase of OA progression. Furthermore, SPG actively safeguarded vital cartilage components such as COL2A1 and ACAN by favorably rebalancing the anabolic-to-catabolic ratio. Taken together, these results provide definitive evidence of the chondroprotective capacity of SPG in OA, encompassing both the effective suppression of catabolism and the dampening of the pathological compensatory anabolic drive.

## Figures and Tables

**Table 1:** Experimental group assignments and dosing regimen.

Group	Substance	Dose (mg/kg/day)	Volume (µL)
Sham	Saline	-	100
Control	Saline	-	100
CLX 20 mg/kg	CLX ^1^	20	100
SPG 50 mg/kg	SPG ^2^	50	100
SPG 100 mg/kg	SPG ^2^	100	100

^1^ CLX, celecoxib. ^2^ SPG, salmon nasal cartilage proteoglycan.

**Table 2:** Primer sequences for quantitative RT-qPCR.

Gene	Forward/Reverse	Sequence (5′→3′)
TNF-α	Forward	AGGGTCTGGGCCATAGAACT
	Reverse	CCACCACGCTCTTCTGTCTA
IL-1β	Forward	CTCGCAGCAGCACATCAACAAG
	Reverse	CCACGGGAAAGACACAGGTAGC
IL-6	Forward	ACAAAGCCAGAGTCCTTCAGAGAG
	Reverse	TTGGATGGTCTTGGTCCTTAGCC
SOX-9	Forward	AGTACCCGCATCTGCACAAC
	Reverse	ACGAAGGGTCTCTTCTCGCT
COL2A1	Forward	GCTGGTGAAGAAGGCAAACGAG
	Reverse	CCATCTTGACCTGGGAATCCAC
ACAN	Forward	CAGGCTATGAGCAGTGTGATGC
	Reverse	GCTGCTGTCTTTGTCACCCACA
MMP-2	Forward	CAAGGATGGACTCCTGGCACAT
	Reverse	TACTCGCCATCAGCGTTCCCAT
MMP-3	Forward	GGCCTGGAACAGTCTTGGC
	Reverse	TGTCCATCGTTCATCATCGTCA
MMP-13	Forward	GATGACCTGTCTGAGGAAGACC
	Reverse	GCATTTCTCGGAGCCTGTCAAC
ADAMTS-4	Forward	ATGGCCTCAATCCATCCCAG
	Reverse	AAGCAGGGTTGGAATCTTTGC
ADAMTS-5	Forward	CTGCCTTCAAGGCAAATGTGTGG
	Reverse	CAATGGCGGTAGGCAAACTGCA
TIMP-1	Forward	TCTTGGTTCCCTGGCGTACTCT
	Reverse	GTGAGTGTCACTCTCCAGTTTGC
TIMP-3	Forward	CACGGAAGCCTCTGAAAGTC
	Reverse	TCCCACCTCTCCACAAAGTT
β-actin	Forward	AGCCATGTACGTAGCCATCC
	Reverse	CTCTCAGCTGTGGTGGTGAA

## Data Availability

Data are contained within the article.

## Supplementary Materials

The following supporting information can be downloaded at: https://www.mdpi.com/article/10.3390/nu18010176/s1, Figure S1: Effect of SPG oral administration on body weight changes in MIA-induced OA mice; Figure S2: Effect of SPG oral administration on body weight changes and hind paw weight-bearing distribution in MIA-induced OA mice.

## References

[1] Woolf A.D., Pfleger B. (2003). Burden of major musculoskeletal conditions. Bull. World Health Organ..

[2] Hawker G.A. (2019). Osteoarthritis is a serious disease. Clin. Exp. Rheumatol..

[3] Hawker G.A., King L.K. (2022). The burden of osteoarthritis in older adults. Clin. Geriatr. Med..

[4] Guccione A.A., Felson D.T., Anderson J.J., Anthony J.M., Zhang Y., Wilson P.W., Kelly-Hayes M., Wolf P.A., Kreger B.E., Kannel W.B. (1994). The effects of specific medical conditions on the functional limitations of elders in the Framingham Study. Am. J. Public. Health.

[5] Creamer P., Lethbridge-Cejku M., Hochberg M. (1999). Determinants of pain severity in knee osteoarthritis: Effect of demographic and psychosocial variables using 3 pain measures. J. Rheumatol..

[6] Chen D., Shen J., Zhao W., Wang T., Han L., Hamilton J.L., Im H.-J. (2017). Osteoarthritis: Toward a comprehensive understanding of pathological mechanism. Bone Res..

[7] De Roover A., Escribano-Núñez A., Monteagudo S., Lories R. (2023). Fundamentals of osteoarthritis: Inflammatory mediators in osteoarthritis. Osteoarthr. Cartil..

[8] Lipari L., Gerbino A. (2013). Expression of gelatinases (MMP-2, MMP-9) in human articular cartilage. Int. J. Immunopathol. Pharmacol..

[9] Rousseau J.-C., Delmas P.D. (2007). Biological markers in osteoarthritis. Nat. Clin. Pract. Rheumatol..

[10] Zhang M., Theleman J.L., Lygrisse K.A., Wang J. (2019). Epigenetic mechanisms underlying the aging of articular cartilage and osteoarthritis. Gerontology.

[11] Poulet B. (2017). Models to define the stages of articular cartilage degradation in osteoarthritis development. Int. J. Exp. Pathol..

[12] Hwang H.S., Kim H.A. (2015). Chondrocyte apoptosis in the pathogenesis of osteoarthritis. Int. J. Mol. Sci..

[13] He Y., Li Z., Alexander P.G., Ocasio-Nieves B.D., Yocum L., Lin H., Tuan R.S. (2020). Pathogenesis of osteoarthritis: Risk factors, regulatory pathways in chondrocytes, and experimental models. Biology.

[14] Charlier E., Relic B., Deroyer C., Malaise O., Neuville S., Collée J., Malaise M.G., De Seny D. (2016). Insights on molecular mechanisms of chondrocytes death in osteoarthritis. Int. J. Mol. Sci..

[15] Akkiraju H., Nohe A. (2015). Role of chondrocytes in cartilage formation, progression of osteoarthritis and cartilage regeneration. J. Dev. Biol..

[16] Poole A.R., Kojima T., Yasuda T., Mwale F., Kobayashi M., Laverty S. (2001). Composition and structure of articular cartilage: A template for tissue repair. Clin. Orthop. Relat. Res. (1976–2007).

[17] Mueller M.B., Tuan R.S. (2011). Anabolic/catabolic balance in pathogenesis of osteoarthritis: Identifying molecular targets. PMR.

[18] Goldring M.B. (2000). The role of the chondrocyte in osteoarthritis. Arthritis Rheum. Off. J. Am. Coll. Rheumatol..

[19] Li M., Xiao R., Li J., Zhu Q. (2017). Regenerative approaches for cartilage repair in the treatment of osteoarthritis. Osteoarthr. Cartil..

[20] Wang Y.-X.J., Griffith J.F., Ahuja A.T. (2010). Non-invasive MRI assessment of the articular cartilage in clinical studies and experimental settings. World J. Radiol..

[21] Pelletier J.-P., Martel-Pelletier J., Rannou F., Cooper C. (2016). Efficacy and safety of oral NSAIDs and analgesics in the management of osteoarthritis: Evidence from real-life setting trials and surveys. Semin. Arthritis Rheum..

[22] Essex M.N., Zhang R.Y., Berger M.F., Upadhyay S., Park P.W. (2013). Safety of celecoxib compared with placebo and non-selective NSAIDs: Cumulative meta-analysis of 89 randomized controlled trials. Expert. Opin. Drug Saf..

[23] Harirforoosh S., Asghar W., Jamali F. (2013). Adverse effects of nonsteroidal antiinflammatory drugs: An update of gastrointestinal, cardiovascular and renal complications. J. Pharm. Pharm. Sci..

[24] Henrotin Y., Mobasheri A. (2018). Natural products for promoting joint health and managing osteoarthritis. Curr. Rheumatol. Rep..

[25] Kang Y.-H., Lee H.J., Lee C.J., Park J.-S. (2019). Natural products as sources of novel drug candidates for the pharmacological management of osteoarthritis: A narrative review. Biomol. Ther..

[26] Kim O.K., Shim T.J., Kim J.H., Lee J. (2014). Effect of Boswellia serrata extracts on degenerative osteoarthritis in vitro and in vivo models. J. Korean Soc. Food Sci. Nutr..

[27] Roshani M., Delfan B., Yarahmadi S., Saki M., Birjandi M. (2025). Impact of olive leaf extract on pain management and functional improvement in elderly patients with knee osteoarthritis: A randomized controlled trial. Explore.

[28] Watanabe F., Bito T., Koseki K. (2025). Salmon meats and by-products as excellent sources of vitamin B12. Fish. Sci..

[29] Abramova L., Kozin A. (2023). Assessment of the Nutrient and Metabolic Profile of the Chum Salmon (*Oncorhynchus keta*). Appl. Biochem. Microbiol..

[30] Elagizi A., Lavie C.J., O’Keefe E., Marshall K., O’keefe J.H., Milani R.V. (2021). An update on omega-3 polyunsaturated fatty acids and cardiovascular health. Nutrients.

[31] Squadrito F., Bitto A., Irrera N., Pizzino G., Pallio G., Minutoli L., Altavilla D. (2017). Pharmacological activity and clinical use of PDRN. Front. Pharmacol..

[32] Hirasawa S., Yabe T. (2025). Salmon Nasal Cartilage Proteoglycan: Exogenous Functionality Compared to Other Chondroitin Sulfates. Rev. Agric. Sci..

[33] Kakizaki I., Mineta T., Sasaki M., Tatara Y., Makino E., Kato Y. (2014). Biochemical and atomic force microscopic characterization of salmon nasal cartilage proteoglycan. Carbohydr. Polym..

[34] Roughley P.J., Mort J.S. (2014). The role of aggrecan in normal and osteoarthritic cartilage. J. Exp. Orthop..

[35] Horkay F., Basser P.J., Hecht A.-M., Geissler E. (2008). Gel-like behavior in aggrecan assemblies. J. Chem. Phys..

[36] Yang B.B., Zhang Y., Cao L., Yang B.L. (1998). Aggrecan and link protein affect cell adhesion to culture plates and to type II collagen. Matrix Biol..

[37] Ono H.K., Yoshimura S., Hirose S., Narita K., Tsuboi M., Asano K., Nakane A. (2018). Salmon cartilage proteoglycan attenuates allergic responses in mouse model of papain-induced respiratory inflammation. Mol. Med. Rep..

[38] Hirose S., Narita K., Asano K., Nakane A. (2018). Salmon cartilage proteoglycan promotes the healing process of Staphylococcus aureus-infected wound. Heliyon.

[39] Sano M., Shang Y., Nakane A., Saito T. (2017). Salmon nasal cartilage proteoglycan enhances growth of normal human dermal fibroblast through Erk1/2 phosphorylation. Biosci. Biotechnol. Biochem..

[40] Asano K., Yoshimura S., Nakane A. (2013). Alteration of intestinal microbiota in mice orally administered with salmon cartilage proteoglycan, a prophylactic agent. PLoS ONE.

[41] Hirose S., Asano K., Harada S., Takahashi T., Kondou E., Ito K., Iddamalgoda A., Nakane A. (2022). Effects of salmon cartilage proteoglycan on obesity in mice fed with a high-fat diet. Food Sci. Nutr..

[42] Kobayashi T., Kakizaki I., Nozaka H., Nakamura T. (2017). Chondroitin sulfate proteoglycans from salmon nasal cartilage inhibit angiogenesis. Biochem. Biophys. Rep..

[43] Lee H.R., Hong S.-M., Cho K., Kim S.H., Ko E., Lee E., Kim H.J., Jeon S.Y., Do S.G., Kim S.Y. (2024). Potential Role of Dietary Salmon Nasal Cartilage Proteoglycan on UVB-Induced Photoaged Skin. Biomol. Ther..

[44] Tomonaga A., Takahashi T., Tanaka Y.T., Tsuboi M., Ito K., Nagaoka I. (2017). Evaluation of the effect of salmon nasal proteoglycan on biomarkers for cartilage metabolism in individuals with knee joint discomfort: A randomized double-blind placebo-controlled clinical study. Exp. Ther. Med..

[45] Takahashi T. (2018). Evaluation of the Efficacy and Safety of Long—Term Intake of a Dietary Supplement Containing Salmon Nasal Cartilage—Derived Proteoglycan on Subjects with Subjective Knee Symptoms—An Open Study—. Jpn. Pharmacol. Ther..

[46] Pitcher T., Sousa-Valente J., Malcangio M. (2016). The monoiodoacetate model of osteoarthritis pain in the mouse. J. Vis. Exp. JoVE.

[47] Nair A.B., Jacob S. (2016). A simple practice guide for dose conversion between animals and human. J. Basic. Clin. Pharm..

[48] Marker C.L., Pomonis J.D. (2012). The monosodium iodoacetate model of osteoarthritis pain in the rat. Pain Research: Methods and Protocols.

[49] Mankin H.J. (1971). Biochemical and metabolic aspects of osteoarthritis. Orthop. Clin. North. Am..

[50] Pauli C., Whiteside R., Heras F., Nesic D., Koziol J., Grogan S., Matyas J., Pritzker K., D’lima D., Lotz M. (2012). Comparison of cartilage histopathology assessment systems on human knee joints at all stages of osteoarthritis development. Osteoarthr. Cartil..

[51] Krenn V., Morawietz L., Burmester G.R., Kinne R., Mueller-Ladner U., Muller B., Haupl T. (2006). Synovitis score: Discrimination between chronic low-grade and high-grade synovitis. Histopathology.

[52] Jang S., Lee K., Ju J.H. (2021). Recent updates of diagnosis, pathophysiology, and treatment on osteoarthritis of the knee. Int. J. Mol. Sci..

[53] Creamer P. (2004). Current perspectives on the clinical presentation of joint pain in human OA. Osteoarthritic Joint Pain: Novartis Foundation Symposium 260.

[54] Chow Y.Y., Chin K.-Y. (2020). The role of inflammation in the pathogenesis of osteoarthritis. Mediat. Inflamm..

[55] Li L., Li Z., Li Y., Hu X., Zhang Y., Fan P. (2020). Profiling of inflammatory mediators in the synovial fluid related to pain in knee osteoarthritis. BMC Musculoskelet. Disord..

[56] Kobayashi M., Squires G.R., Mousa A., Tanzer M., Zukor D.J., Antoniou J., Feige U., Poole A.R. (2005). Role of interleukin-1 and tumor necrosis factor α in matrix degradation of human osteoarthritic cartilage. Arthritis Rheum. Off. J. Am. Coll. Rheumatol..

[57] Porée B., Kypriotou M., Chadjichristos C., Beauchef G., Renard E., Legendre F., Melin M., Gueret S., Hartmann D.-J., Malléin-Gerin F. (2008). Interleukin-6 (IL-6) and/or soluble IL-6 receptor down-regulation of human type II collagen gene expression in articular chondrocytes requires a decrease of Sp1· Sp3 ratio and of the binding activity of both factors to the COL2A1 promoter. J. Biol. Chem..

[58] Jeong I., Park J., Park S., Wada T., Lim D.S., Kim O.-K. (2024). Salmon Nasal Cartilage-Derived Proteoglycans Alleviate Monosodium Iodoacetate-Induced Osteoarthritis in Rats. Mar. Drugs.

[59] Kim H., Kang D., Cho Y., Kim J.-H. (2015). Epigenetic regulation of chondrocyte catabolism and anabolism in osteoarthritis. Mol. Cells.

