# Supplementary material for: Salmon Nasal Cartilage Proteoglycan Ameliorate Joint Pain and Cartilage Degradation by Regulating Catabolic and Anabolic Homeostasis in MIA-Induced Osteoarthritis
Source: Nutrients. 2026 Jan 5;18(1):176. doi: 10.3390/nu18010176 (PMC12788049; doi:10.3390/nu18010176)
Supplement: Supplementary file 1 [file nutrients-18-00176-s001.zip › nutrients-4047531-supplementary.pdf]

Supplementary Figures

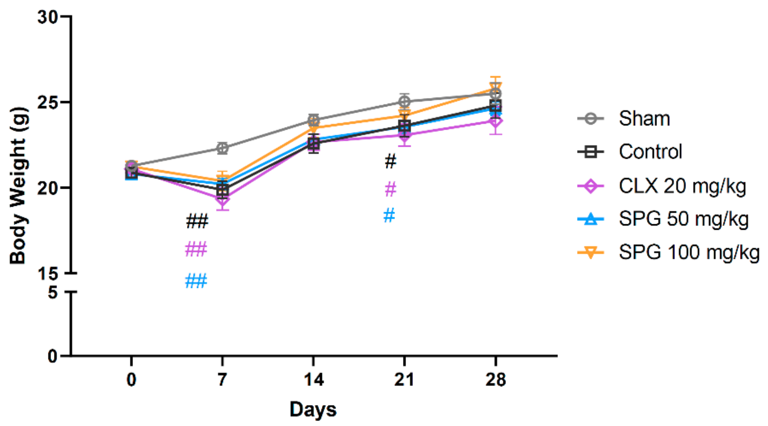

Supplementary Figure S1. Body weight changes in experimental groups of the cohort for pain assessment and histological analysis. Body weight of the mice (cohort #1) used for pain assessment, histology and immunohistochemistry was measured on every 7 days until Day 28. Data are presented as the mean ± SEM (*n* = 10 - 12 per group). Statistical significance was determined using repeated-measure (RM) Two-way ANOVA followed by Dunnett's post hoc test. Significant differences are indicated as follows: # $p<0.05$ *vs*. Sham; and ## $p<0.01$ *vs*. Sham. CLX, Celecoxib; SPG, Salmon nasal cartilage proteoglycan.

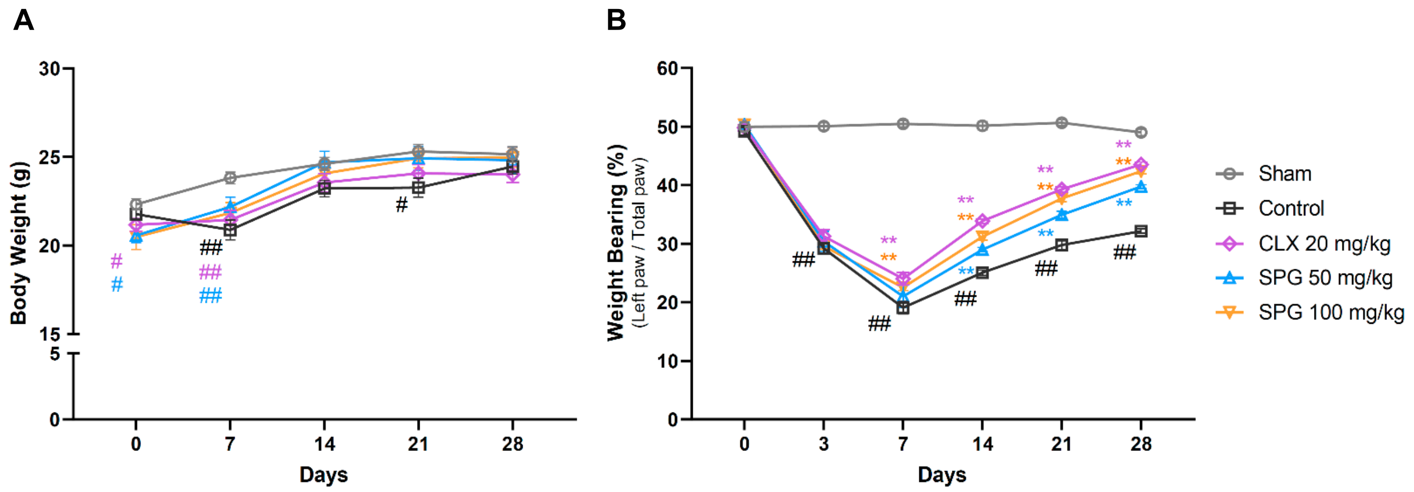

Supplementary Figure S2. Body weight changes and hind paw weight-bearing distribution in experimental groups of the cohorts for mRNA expression analysis. Body weight **(A)** and the hind paw weight-bearing distribution **(B)** of the mice (cohorts #2 and #3) used for the mRNA expression analysis were measured every 7 days until Day 28. Data are presented as the mean ± SEM (*n* = 20 - 22 per group). Statistical significance was determined using repeated-measure (RM) Two-way ANOVA followed by Dunnett's post hoc test. Significant differences are indicated as follows: ## $p<0.01$ *vs*. Sham; and ** $p<0.01$ *vs*. Control. CLX, Celecoxib; SPG, Salmon nasal cartilage proteoglycan.
